# ViVar: A Comprehensive Platform for the Analysis and Visualization of Structural Genomic Variation

**DOI:** 10.1371/journal.pone.0113800

**Published:** 2014-12-12

**Authors:** Tom Sante, Sarah Vergult, Pieter-Jan Volders, Wigard P. Kloosterman, Geert Trooskens, Katleen De Preter, Annelies Dheedene, Frank Speleman, Tim De Meyer, Björn Menten

**Affiliations:** 1 Center for Medical Genetics, Faculty of Medicine and Health Sciences, Ghent University, Gent, Belgium; 2 Department of Medical Genetics, University Medical Center Utrecht, Utrecht, The Netherlands; 3 BioBix, Faculty of Bioscience Engineering, Ghent University, Gent, Belgium; Swiss Institute of Bioinformatics, Switzerland

## Abstract

Structural genomic variations play an important role in human disease and phenotypic diversity. With the rise of high-throughput sequencing tools, mate-pair/paired-end/single-read sequencing has become an important technique for the detection and exploration of structural variation. Several analysis tools exist to handle different parts and aspects of such sequencing based structural variation analyses pipelines. A comprehensive analysis platform to handle all steps, from processing the sequencing data, to the discovery and visualization of structural variants, is missing. The ViVar platform is built to handle the discovery of structural variants, from Depth Of Coverage analysis, aberrant read pair clustering to split read analysis. ViVar provides you with powerful visualization options, enables easy reporting of results and better usability and data management. The platform facilitates the processing, analysis and visualization, of structural variation based on massive parallel sequencing data, enabling the rapid identification of disease loci or genes. ViVar allows you to scale your analysis with your work load over multiple (cloud) servers, has user access control to keep your data safe and is easy expandable as analysis techniques advance. URL: https://www.cmgg.be/vivar/

## Introduction

Structural variations (SVs) play an important role in genetic diversity and are responsible for many human genetic disorders [1], [2]. In recent years, genomic microarrays have been invaluable in the elucidation of structural variation in both patient and normal control samples [3]–[5]. Genomic microarrays uncovered copy number variation (CNV) as an important source for genomic variation in addition to single nucleotide variants (SNVs) and genomic microarrays accelerated the discovery of novel disease causing CNVs. The recent implementation of novel high-throughput sequencing technologies provide new and powerful alternatives for genomic microarrays for the detection of CNVs [6]–[8]. These technologies have several advantages over genomic microarrays; copy number variations (CNVs) can be detected at ultra-high resolution, down to the base-pair level. Moreover, they enable the rapid elucidation of the genomic architecture of duplications or insertional translocations and unlike microarrays, they are able to detect both unbalanced as well as balanced rearrangements.

The introduction of these new technologies also poses new challenges regarding data analysis and interpretation. Current genomic sequencers produce massive amounts of data that can only be interpreted after intelligent processing and filtering. The ultimate goal is to distill these huge amounts of sequence reads to a set of clinically relevant structural variants.

To facilitate data processing, interpretation and visualization of sequencing and/or genomic microarray data, we developed the ViVar platform. By uploading raw sequencing reads or genomic microarray data to the ViVar server, the platform will run the appropriate processing pipeline, including coverage and discordant readpair clustering analyses. Once the data is processed, results are available for further downstream visualization and interpretation within the ViVar platform, such as a summary report, a virtual karyogram and a zoomable annotated genome browser.

## Materials and Methods

The goal is to serve all types of users within a research or diagnostics setting (lab technicians, medical doctors and researchers) without the need of extensive bioinformatics training or having to gain experience with each of the different analysis components itself. Once the platform is deployed on a webserver, it can be accessed using a recent web browser (no plugins are required). After uploading sequence data, all raw data-processing is handled in the background with preset, adjustable parameters. It uses well-established, published tools as components in the pipeline, while retaining the flexibility for advanced users to adapt the platform and underlying tools to specific needs (R [9], CBS [10], bwa [11], [12], samtools [13], GATK [14], CNV-Seq [15], BreakDancer [16], Picard [17], MongoDB [18] and sqlite [19]).

### Discovery of structural variants

The analysis pipeline is optimized for mate-pair/paired-end sequencing data, detecting large and small CNVs but also balanced structural variants such as inversions, insertions, translocations and complex rearrangements. In recent years, several algorithms have been published to extract structural variants (SVs) from next generation sequencing data, each with its specific strengths and weaknesses and the comparison study by Duan et al [20] highlights the need for improvement of the different algorithms. Many of these tools are hard to implement for geneticists, while the output of these algorithms is often hard to compare or interpret. With ViVar, a list of SVs is generated by integrating, optimizing and complementing depth of coverage analysis (DOC), aberrant read pair clustering and split read analysis. While most examples in this paper will use human data, all methods can be applied to other organisms for which a good reference genome is available. Besides human samples, ViVar currently supports analyzing samples from common experimental organisms such as mouse (Mus musculus) and zebrafish (Danio rerio).

### Preparation of reads

ViVar employs the well-established short read mapping algorithms bwa [11], [12], bowtie [21], Stampy [22] or SSAHA2 [23] to align the reads to the reference genome. PCR-duplicates that can arise during the amplification steps of the library preparation are filtered out using Picard-tools (should and can be disabled when using a transposase or PCR amplicon based library preparation method, which generates false positive read duplicates).

### Depth of coverage (DOC) analysis

The first approach to find structural variants is to analyze the depth of coverage (DOC) for each sample in the dataset. In this analysis, we employ the CNV-seq algorithm [15]. The number of mapped reads is counted in bins using sliding windows along the chromosomes, and for each window/bin, a coverage ratio between the sample and a reference set is calculated to compute a predicted copy number ratio and its probability. The size of the windows/bins is determined by the overall coverage of the sample and the genome size under investigation. Read mapability, platform specific variation and GC-content influence coverage, making the choice of a good reference set essential. This reference set should ideally be composed of several normal samples not containing any CNV of interest. The data generated for this reference should preferably be generated according to the same or similar experimental conditions as the tested sample, allowing correction for sequencing platform and library preparation specific characteristics.

### Aberrant read pair clustering

The second approach for SV evaluation, complementing the DOC analysis, is the clustering step. This algorithm is implemented as a hard-clustering solution to group similar pairs, indicating a single structural variant. The SV is represented as a cluster of discordant pairs with similar characteristics derived from the read mapping information, i.e. mates/pairs featured by larger than expected insert sizes ( =  distance between two reads of a pair) or unexpected orientation.

After successful mapping, the insert size distribution is build and the median insert size and standard deviation are calculated to set the thresholds for the identification of discordant pairs. To discard false discordant pairs due to mapping artifacts, a local realignment is attempted using ClustalW-powered realignment for all aberrant pairs [24].

**Figure 1:**
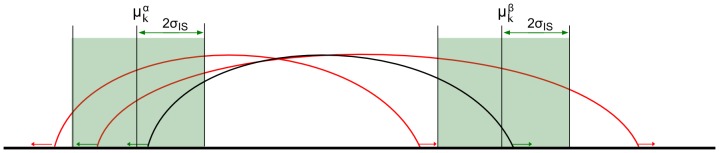
Clustering. Illustration of the criteria to determine similarity when grouping pairs in clusters: pair *i* is considered to be part of a cluster if the start position of the first (α_i_) and second read (β_i_) of the pair is within a region centered on the mean start position of all reads in start (µ^α^_k_) and end (µ^β^_k_) of the cluster *k* and extended left and right with twice the standard deviation of the mean insert size calculated for all pairs in the sample (σ_IS_). Pair *i* is member of cluster *k* **if** 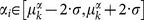 ******and****** 
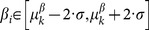
.

The remaining discordant pairs are clustered, according to their genomic position, in groups covering the same structural variant. This hard-clustering algorithm loads all uniquely mapped mates/pairs and compares them with the existing clusters. While scanning the sorted list of sequencing reads, a cluster will be build when 2 or more sets of mates/pairs have similar genomic locations for their first and second read and have a similar insert size. Fig. 1 illustrates the similarity criteria for reads to be grouped in the same cluster, and shows the overlapping region formed between the start and stop of a cluster. This region will contain the potential breakpoint site. Mates/pairs can only be part of one cluster, being the closest matching one. If no matching cluster is found, the mates/pair will form a new cluster. When all reads have been processed, based on the coverage data, a minimal read count cut-off value is set to filter out clusters containing an insufficient amount of similar pairs, and thus not providing enough evidence to indicate a plausible breakpoint. The variant type of a cluster can be assigned based on the read signature caused by each structural variant [25]. Detection of simple duplications, inversions, deletions, insertions and translocations is automated, but complex variants require manual interpretation of the cluster pattern.

Besides the in-house developed clustering method described in the previous paragraph, the BreakDancer algorithm was included [16], which implements similar methods of using discordant reads to predict SVs.

### Split read analysis

The third approach integrated in ViVar to detect structural variants is a split read analysis using Pindel [26]. By means of a pattern growth approach, Pindel searches for indels within the reads themselves.

Variants discovered with DOC analysis, clustering and Pindel are combined, and various filter steps can be applied to minimize the number of false positive results. Variants are evaluated for their overlap with segmental duplications, RepeatMasker regions or if they coincide with the hg19 Self Chain record (UCSC data tables [27]). These steps eliminate technical artifacts due to aberrant mapping in repetitive regions.

### Genomic microarray data analysis

Besides structural information from sequencing, ViVar also includes a genomic microarray data analysis pipeline. Input from different providers (Agilent, Affymetrix or Illumina) can be used to survey copy number changes on a genome wide scale. The raw fluorescence intensity ratios are segmented with the CBS algorithm [10]. From this, a list of regions with copy number gain or loss can be obtained. Furthermore, ViVar supports the analysis of SNP arrays. The genotyping information can be used to locate regions with loss of heterozygosity (LOH) or to search for identical by descent (IBD) regions in consanguineous families using the integrated PLINK algorithm [28]. As such, ViVar allows the investigator to combine the latest sequencing based technologies with the existing golden standard for copy number analysis.

### Visualization

ViVar is a web based platform for which we use the latest HTML5 standards combined with JavaScript to build a dynamic interface. We use scalable vector graphics to render the visualization, which is resolution independent, thereby obtaining optimal image quality (even on high resolution screens).

### Computing platform

Particular attention was paid to the use of open source software for the development of the ViVar platform. The platform is Ubuntu linux based, and uses Perl and PHP scripting languages, Nginx webserver, R based statistics and MongoDB, a document based database store. Detailed installation instructions can be found on the website. While all user interactions are handled through the web interface, the analysis server requires significant resources to be able to handle the many computational and memory intensive tasks contained in the pipeline. To meet that challenge, we designed ViVar to be deployed on a cloud platform. Long running analysis tasks can be submitted to a work queue system for optimal workload distribution.

## Results and Discussion

### Usability & Scalability

The ViVar tool is a web based data analysis and visualization platform. We aim to complement and extend, and not reinvent, existing analysis tools. The focus is on better usability of the complex underlying algorithms during data analysis, and integrating the produced results directly with our novel visualization layer. By virtue of the web interface, the tool is accessible to a broad range of users without precluding advanced users to control all analytical steps with a configurable back-end. The computing platform is designed so that if datasets continue to grow as sequencing technology advances, this will allow users to easily expand the database to new virtual instances and scale the back-end as needs increase.

### Visualization

Visualization of processed data is essential since it facilitates the interpretation and manual curation of results, especially since sequencing data can provide evidence for very complex rearrangements that are difficult to unravel without proper visual inspection. Existing genome browsers, such as the UCSC Genome Browser, IGV and GBrowse, support basic visualization of structural variants, but are limited to simple (colored) line segments plotted along a linear genome representation. ViVar takes full advantage of the modern web browsers and HTML standards to deliver a powerful dynamic interface, supporting a wide range of visualizations to explore the data.

**Figure 2:**
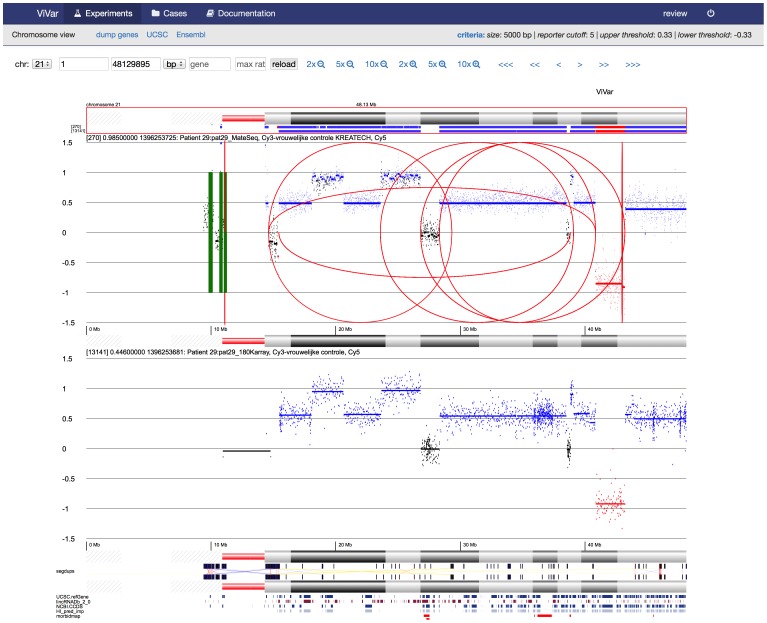
Chromosome view, a patient with a complex trisomy 21. (top) sequencing data based coverage and clustering information (bottom) genomic microarray profile. Aberrant clusters are depicted as red arches. Horizontal segments delineate coverage windows in case of sequencing data or microarray probes in case of genomic-microarray/arrayCGH data, segment can be colored in blue when indicating a gain or red for a loss. Below the ratio, cluster plot of the samples, 2 chromosome arms are draw with segmental duplications shown between them. The lower part contains the annotation tracks.

The main visualization type is a linear genome browser, named “chromosome view”. The browser uses a zoomable window to explore the data at different resolution levels, and allows easy comparison of multiple samples (Fig. 2). Besides the traditional genome viewer, a circular view is available to facilitate comprehension of intra- and inter chromosomal rearrangements.

The experimentally obtained data can be complemented with a multitude of annotation tracks to guide the interpretation. Segmental duplications and human chained self-alignments provide information on the underlying genomic architecture, the RefSeq and CCDS track places the data in its local genetic context, the OMIM morbid/gene-map and the Database of Genomic Variants [29] track provide a biological/clinical context. As the original sources of this external annotation data undergo frequent updates, ViVar only performs updates when explicitly requested. Because of this, users can keep the annotation versions stable/unchanged and trust to have reproducible results when using unchanged filter parameters, this can be important in some cases i.e. when reporting on clinical samples. The annotation track system can be used to build your own tracks, e.g. a track for comparing individual results with existing data collections of experiments to find correlations in your internal datasets.

**Figure 3:**
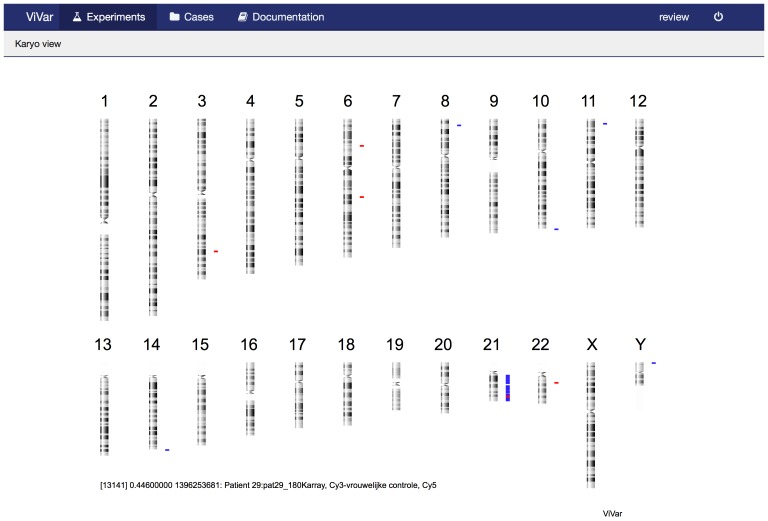
Karyoview, showing a karyogram of a patient with a complex trisomy 21.

**Figure 4:**
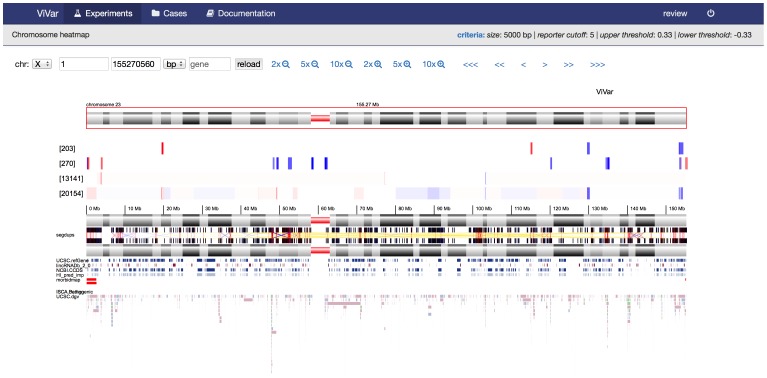
Heatmap, plotting copy number variants for two sequencing and two genomic microarray based experiments.

To get a high level overview, the “karyoview” reduces the data to the most significant variants and draws these on a karyogram (Fig. 3). Multiple samples can be visualized together to explore data in its sample set context. ViVar provides multiple sample support in the “chromosome view” and “karyoview”. Additionally, a “heatmap” view plots the coverage ratio in a heatmap for a set of samples making it easier to visualize recurrence patterns in large datasets (Fig. 4).

If further visualization is needed, users can export the produced alignments as a bam-file and variant lists as a txt file (VCF format), and use other available visualization methods aimed at visualization of SVs (Meander [30], fastbreak [31], Gremlin [32]), or experienced users can write an add-on to directly incorporate one of these in ViVar.

### Reporting

**Figure 5:**
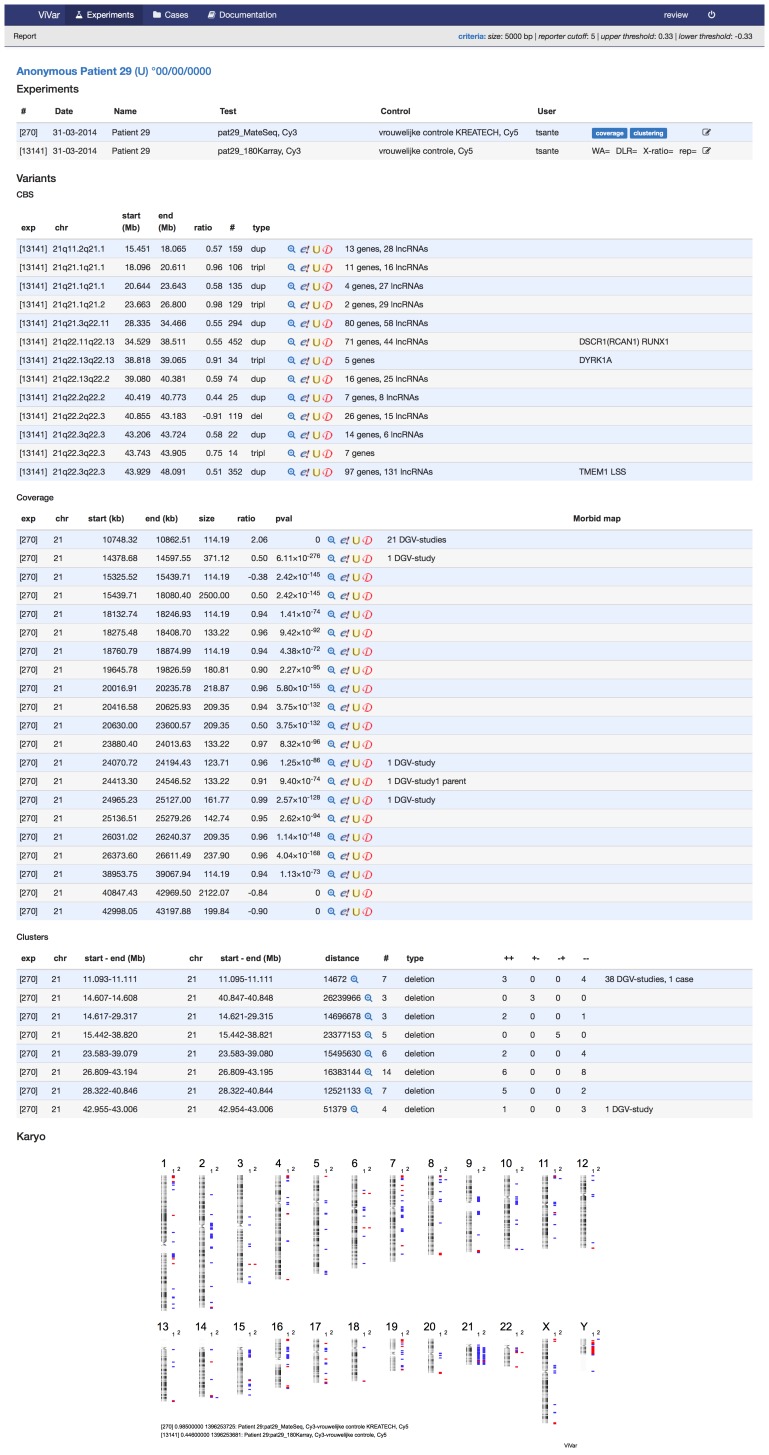
Report. View an overview of all experiments for a case/patient with a complex trisomy 21.

Besides analysis and visual inspection of the data, ViVar generates a comprehensive report of a sample, combining all available data in the platform (Fig. 5).

The first component of this report summarizes the sample annotation, such as parental information, date of birth and/or clinical information. The next component is an overview of all experiments for a specific sample with associated experiment-related information. A third part of the report contains a table with detected structural variations. These SVs can be filtered on the number of evident reporters/clusters or size of the aberration. Each listed variant is linked to external and internal databases: OMIM annotation, gene overlap (CCDS, RefSeq, UCSC), lncRNAs [33], the Database of Genomic Variant studies (DGV) [29] and recurrence in external or internal sample sets. The table also includes shortcuts to the focused genomic location in the different visualizations of the specific aberration.

Finally the ”karyoview”-component of the report displays a visual overview of the aberrations listed in the table. These three components combined in one report, distill your data into a handy format, ideal to get a quick summary of the results as a starting point of an in depth study, or as a report for diagnostic use.

### Data management

Good data management facilitates record keeping and traceability of all experiments. As such, each experiment can be coupled to a case (or sample) and annotated with all necessary information about the experiment e.g., library design, array design, data files, and operator. To organize experiments, they can be grouped into projects and shared between users and projects. All information is stored in a NoSQL, document-based database and thus easy to search.

The platform is secured by an administrator account that can assign fine-grained access restrictions. By default a user can only access his/her own data, but can be given access to case annotation, experiment groups and even individual experiments. For each access level, a user can be assigned a read-only role or given full edit rights. Significant changes to existing data can be logged to allow for traceability compliance, ensuring that data is not only easily manageable but also securely stored.

### Validation

ViVar was tested on a validated set of 50 patient samples. The sequencing data was generated using mate pair sequencing on a SOLiD sequencer (Life technologies) and HiSeq 2000 (Illumina) [6]. Simultaneously, all samples were analyzed by high-resolution arrayCGH analysis and conventional karyotyping. ViVar analysis enabled the rapid identification of all variants detected by arrayCGH analysis and karyotyping. DOC analysis, clustering and indel analysis proved to be complementary, maximizing the detection rate of structural variants.

## Conclusions

ViVar is a user friendly and easy to implement comprehensive analysis, visualization and data management platform for mate-paired/paired end sequencing data and genomic microarray experiments. This tool can greatly facilitate identification of structural variants from massive amounts of sequencing data. By bringing together several validated analysis tools in one platform and providing an integrated visualization module, users no longer face the difficulty of manually running each separate analysis step, but are still empowered to adopt underlying tools for their specific needs.
